# A Treatise on the Venereal Disease

**Published:** 1859-04

**Authors:** 


*A Treatise on the Venereal Disease.* By JOHN HUNTER, F. R. S. With copious additions, by Dr. Philip Ricord, Surgeon of the *Hôpital du Midi*, Paris, etc. Translated and Edited, with Notes, by Freeman J. Bumstead, M. D., Lecturer on Venereal at the College of Physicians & Surgeons, N. Y.; Assistant Surgeon to the Eye Infirmary. Second Edition, Revised, containing a *Résumé* of Ricord's Recent Lectures on Chancre. Philadelphia: BLANCHARD & LEA. 1859.

This work is too well known to the Profession, to necessitate anything more than the simple announcement of a new edition. To the former edi_ tion, have been added translations of the new points contained in Ricord's late lectures on Chancre, published from the notes of M. Fournier, Interne of the *Hôpital du Midi*; presenting, in the present volume, John Hunter's great work on the Venereal; with Notes by Sir Everard Home; the notes and additions of M. Ricord, and the notes of Mr. Babington, translated and edited by Dr. Bumstead of New York. This makes an octavo of over five hundred pages, which is illustrated by eight elegant plates; a book which is needed by every practitioner.

---

*Inhalation of Carbonic Acid as an Anæsthetic.* (From the French of Dr. OZANAM).

The effects of carbonic acid resemble those of ether, according to the author, but are more fugitive; and while it is necessary in the case of ether to interrupt the inhalations after short intervals, an opposite procedure is required for carbonic acid.

*a.* As long as one wishes the sleep to be prolonged, the inhalations must be continued.

*b.* These can be prolonged ten, twenty, thirty minutes and more, without danger to life.

*c.* When the inhalations are stopped, the waking is almost always immediate.

The experiments of Ozanam and Faure have never resulted in death. When death does take place, it is slow, progressive, and one can predict for some time in advance the moment of its arrival, by considering, as Faure has done, the condition of the heart and the pupils. The following experiment, related by Ozanam, is most interesting.

I had prepared by Mons. Fontaine a gas bag containing about 100 litres of carbonic acid, being resolved to prolong the anæsthesia as far as possible. The animal was put to sleep in three minutes, without convulsions, and remained on its side in a quiet sleep without being held. The inhalations were continued for 87 minutes; and the apparatus was then withdrawn; full sleep lasted five minutes more; towards the tenth minute, the paws began to be agitated; at the fifteenth the animal arose. One hundred and two minutes were thus consumed in the experiment—a time much longer than is required by the longest operations.

We believe that Faure and Ozanam propose the use of *asphyxiated anœsthesia, or anœsthesia produced by carbonic acid,* for man. Faure and Ozanam say, that they have respired the gas, if not to the point of producing sleep, at least until they felt the first effects. Its taste is slightly piquant, about as pleasant as that of ether, and it is an excitor of the saliva. Ozanam says that the ethers, chloroform, and carbonic oxide determine anæsthesia by robbing the arterial blood of its oxygen, so as to produce carbonic acid, and thus making the blood venous. Carbonic acid itself does not decompose the blood; it removes no vital principle from it, but contributes progressively, and so that it can be graduated at will, the necessary quantity of carbon to determine the insensibility.—*American Med. Monthly.*

---

*The Mütter Museum.*—It may be remembered, that when failing health compelled Dr. Thomas D. Mütter, the distinguished professor of surgery in the Jefferson Medical School, to withdraw from his elevated position as a teacher and surgeon, he proposed to endow the Philadelphia College of Physicians with his valuable museum.

Owing to some unforeseen delay, this intention has but recently been carried into effect. The College of Physicians have, however, accepted the donation of Dr. Mütter, who has also munificently added a fund of *thirty thousand dollars*, the interest on which is to be appropriated to the institution of a yearly course of lectures, to be delivered before the College of Physicians. The museum will be deposited in a fire-proof building, to protect it from injury, and the name of the liberal donor will be commemorated in every recurring series of the "Mütter Lectures."—*Virginia Med. & Surg. Jour.*

*Respiratory Movements of the Infant in the Uterus, perceptible by Auscultation.* By Dr. B. SCHULTZE, of Berlin. Translated from the Gazette Médicale.

The attempts at respiration of the infant in the uterus when the placenta is detached, are well known, but these movements have not yet been proven by the stethescope. The author having been called to a woman in labor for the fourth time, found the cord prolapsed. During his attempts to reduce and pass the cord above the head of the fœtus, he felt movements of the child, throwing his head backwards, and opening its mouth at regular intervals. M. Schultze retained the hand in the uterus, placed his ear over this region, and heard between the sounds of the heart, a gurgling noise, similar to that which is sometimes heard in the intestines, but coinciding with the movements of the mouth. The infant was born asphyxiated, and epidermic cells, fine hair, and meconium, coming from the amniotic fluid, werefound in the air passages, evidently inhaled by the efforts at respiration.—*Savannah Journ. of Med.*

---

*The "Gorilla."*—The London Morning Post describes a peculiar zoölogical novelty which has been received at the British Museum, and by the trustees of that institution, loaned to the Crystal Palace for exhibition. It is a specimen of the *Gorilla*, an animal said more nearly to approach the human species than any yet discovered, and whose existence was long deemed fabulous. Ten years ago a traveler in the interior of Africa was struck by seeing the natives worshipping what seemed to be a human skull, set on a pole. With some trouble, the traveler gained possession of the idol, and sent it to London to be examined by Professor Owen. He read his views on the subject in an interesting paper to the Zoölogical Society, deciding that the animal to whem the skull belonged, was not a member of any known family of Chimpanzees or monkeys, and that it was altogether unrepresented by any specimen of natural history known to the scientific world. His drawings of the restored animal were ridiculed by many as fanciful, but at last they are confirmed, and the accuracy of scientific observation proved. An animal was caught after great exertions, packed in spirits of wine and sent to London, and it *coincides in every respect with the animal Prof. Owen had figured from the skull only.* It is a native of Western Africa, and is said to exist in large numbers in the Gaboon districts, where it is among the most formidable of the beasts of the forest. The specimen has been skinned and preserved. It is rather more than five feet high, a male, known to be young, from the state of its teeth and the condition of the sutures of the skull. The fore legs or arms are of great length and strength, far surpassing the human; the hind legs are very short, adapted for tree climbing. In features, it is very like the negro, and the orbits are considerably projected. The teeth are formed precisely as in man, and are of enormous strength, the canine teeth of the first skull sent being as large and strong as those of a lion. The negroes of the country live in constant terror of these Gorillas, which are gregarious, and they state that the animals frequently descend in considerable force, sack the villages, carry off the young children and devour them; also that they have an ugly custom of attacking men, and wrenching off the heads of those they attack. If one of the creatures is fired at or provoked, the whole tribe comes down to the rescue, and escape from the combined assault is impossible.—*Nashville Journal of Medicine and Surgery.*

---

*Death from Want of Sleep.*—The question, how long a person can exist without sleep is one oftener asked than answered, and the difficulties would seem to leave it forever unsolved. A communication to a British Society has, so far as it relates to one instance, seemed to answer the inquiry, in a description of a cruel mode of punishment peculiar to the Chinese. A Chinese merchant had been convicted of murdering his wife, and was sentenced to die by being deprived of sleep. This painful mode of death was carried into execution under the following circumstances:—The condemned was placed in prison under the care of three of the police guard, who relieved each other every alternate hour, and who prevented the prisoner from falling asleep, night or day. He thus lived for nineteen days without enjoying any sleep. At the commencement of the eighth day his sufferings were so intense that he implored the authorities to grant him the blessed opportunity of being strangulated, guillotined, burnt to death, drowned, garroted, shot, quartered, blown up with gunpowder, or put to death in any conceivable way which their humanity or ferocity could invent. This will give a slight idea of the horrors of death from want of sleep.—*Louisville Med. News.*

